# Perceptual decisions are biased toward relevant prior choices

**DOI:** 10.1038/s41598-020-80128-0

**Published:** 2021-01-12

**Authors:** Helen Feigin, Shira Baror, Moshe Bar, Adam Zaidel

**Affiliations:** The Gonda Multidisciplinary Brain Research Center, Bar Ilan University, 5290002 Ramat Gan, Israel

**Keywords:** Neuroscience, Psychology

## Abstract

Perceptual decisions are biased by recent perceptual history—a phenomenon termed 'serial dependence.' Here, we investigated what aspects of perceptual decisions lead to serial dependence, and disambiguated the influences of low-level sensory information, prior choices and motor actions. Participants discriminated whether a brief visual stimulus lay to left/right of the screen center. Following a series of biased ‘prior’ location discriminations, subsequent ‘test’ location discriminations were biased toward the prior choices, even when these were reported via different motor actions (using different keys), and when the prior and test stimuli differed in color. By contrast, prior discriminations about an irrelevant stimulus feature (color) did not substantially influence subsequent location discriminations, even though these were reported via the same motor actions. Additionally, when color (not location) was discriminated, a bias in prior stimulus locations no longer influenced subsequent location discriminations. Although low-level stimuli and motor actions did not trigger serial-dependence on their own, similarity of these features across discriminations boosted the effect. These findings suggest that relevance across perceptual decisions is a key factor for serial dependence. Accordingly, serial dependence likely reflects a high-level mechanism by which the brain predicts and interprets new incoming sensory information in accordance with relevant prior choices.

## Introduction

Perceptual decisions are not based solely on current sensory information. Rather, they are substantially influenced by other factors, such as context and prior experience^1–5^. Notably, our perceptual decisions are biased by preceding perceptual events—a phenomenon, termed ‘serial dependence’, that has garnered considerable recent interest^6–10^, and which suggests that previous goal selections and outputs are actively tracked by the brain to influence subsequent actions^11^. Serial dependence can aid or impair performance, depending on circumstances. For example, when a discriminated feature is distributed independently over time (or across trials, as is often the case in laboratory experiments) preceding information might have little bearing on subsequent stimuli, and therefore serial dependence may add noise and can degrade performance^12^. By contrast, events in the real world are generally not independent in that they follow prototypical regularities in time and space. Hence, serial dependence might benefit everyday performance by supporting perceptual stability over time^6,13,14^, and/or by improving speed and efficiency of perceptual decisions^15–17^. Overall, serial dependence, like predictions more generally, likely reflects a trade-off between efficiency (relying on expectations) and accuracy (relying on instantaneous sensory information).

Many factors may trigger serial dependence, including sensory inputs^6,18^, motor responses^19,20^, expectations^21,22^ and spatial attention^6,23^. It has even been proposed that prior choices, in and of themselves, can elicit serial dependence^24,25^. However, the nature of the elements that underlie serial dependence and their relative contribution to this phenomenon are not fully known.

Here we reasoned that if serial dependence is harnessed by the brain to aid function and efficiency^11^, then it should only be applied when prior perceptual events are considered *relevant* to the impending perceptual decision. By “relevant”, we mean that the observer may implicitly presume that similar, consecutive, stimuli are statistically related, and therefore prior events can aid subsequent perceptual decisions^26^. Conversely, when prior stimuli are presumed *irrelevant* to the current perceptual decision, these should elicit little or no serial dependence. Note that presumed relevance (or irrelevance) is a subjective state—inferred by the observer—and may or may not be in line with the objective state (which is unknown to the observer).

In support of this idea, it was recently found that estimates of a stimulus feature (variance) were biased only when that same feature was discriminated, but not when a different feature (mean), was discriminated^27^. However, others^28,29^ did find serial dependence when participants were told that prior stimuli were irrelevant and were asked to ignore them. Therefore, the question of relevance in serial dependence, as well as how it may interact with other sensory and action-related factors requires further investigation, and it is the aim of our study.

In line with our aim, we hypothesized that relevance between sequential perceptual events, may trigger serial dependence, and propose that when a specific feature of interest is sampled and discriminated across multiple presentations of a stimulus, people would incorporate prior information from their recent choices.

To test this hypothesis, we devised a novel paradigm of visual location discrimination that induces short-term biases over several (prior) stimuli and tests their effects in an interleaved and balanced manner. We manipulated specific features of the prior decisions, including stimulus characteristics, which feature was discriminated, and the motor action used to report choices. We refer to the process of discriminating a stimulus feature as a *perceptual decision*, and the result of the process as the *choice*, which may (or may not) be reported via a motor response.

We found that recent perceptual decisions elicit serial dependence only when relevant choices were made. This was seen even when the prior choices were reported via different motor actions, and when the prior stimuli had different sensory attributes from the test stimuli. As long as the same feature (location) was being discriminated in both prior and test stimuli, serial dependence was observed. By contrast, irrelevant prior choices (about stimulus color) did not bias subsequent location discriminations, even when the prior motor actions or prior stimulus locations (which were not discriminated) were biased. Additionally, even though low-level features did not elicit substantial serial dependence by themselves, greater similarity of low-level features boosted serial dependence. Thus, relevance across sequential decisions may be a key element for serial dependence.

## Methods

### Participants

A total of 115 participants took part in the study (68 females; mean age ± SD: 24 ± 3.54, range: 18–37 years). All participants were healthy and had normal or corrected-to-normal vision. This study was approved by the Bar-Ilan University, Gonda Brain Research Center Ethics Committee, and was performed in accordance with the guidelines and regulations under the given ethical approval. All participants signed informed consent and received monitory compensation for their participation. The data gathered were pre-screened to confirm task understanding and cooperation (see *Statistical analysis* section below for details). This excluded 11 participants’ data, leaving 104 for further analysis. Additional participant details, pertaining to specific experimental conditions, are presented below in the section “Experimental conditions”.

### Experimental design

Stimuli were generated and presented using PsychoPy software^30^ on an LCD monitor with a resolution of 1920 × 1200 pixels and a refresh rate of 60 Hz. Experiments were run in a dark, quiet room. Participants were seated in front of the monitor, with their head positioned 45 cm from the center of the screen and supported by a chin rest. Stimuli comprised uniformly filled circles (6.36° visual angle) presented around the center of the screen on a dark gray background. Circle location was varied in the horizontal dimension only. Participants performed a two-alternative forced choice (2AFC) task in which they discriminated the location of the circle presented (left or right relative to the screen’s center) by pressing the corresponding arrow key on a standard computer keyboard. Participants were instructed to make their choice as rapidly and accurately as possible. They were informed that task difficulty varies with circle proximity to the screen center and instructed to make their best guess when in doubt.

**Figure 1:**
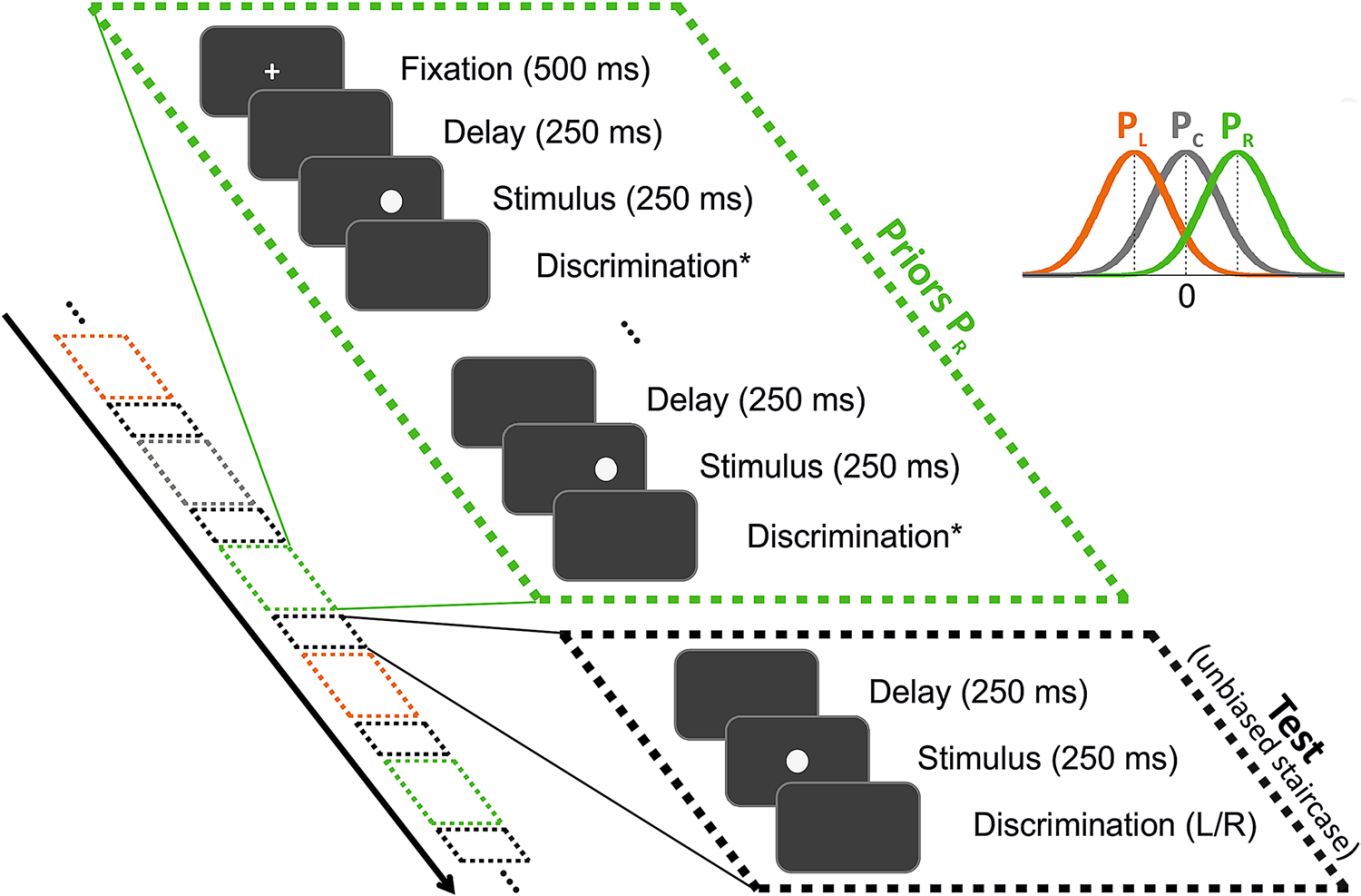
Event sequence within and across trials. Each trial began with a fixation point (at the screen center) followed by a series of discriminations of ‘prior’ stimuli (for example, priors biased to the right, green dashed boxes), and then discrimination of a single ‘test’ stimulus (black dashed boxes). The black arrow marks the time-course of the experiment, which interleaved trials of different prior types pseudo-randomly (right, left and center, marked by green, orange and gray boxes, respectively), each ending with a test stimulus (black box). The stimuli were circles on a dark gray screen, and were discriminated individually (in order). The discrimination task for the test circles was about location: was the circle to the left or to the right of the screen center? For the prior circles, the discrimination task (*) depended on the experimental condition. It was either location discrimination (like the test task) or color discrimination (unlike the test task). All discriminations required choosing one from two alternatives: right/left or green/purple (for location and color discrimination, respectively). Prior circle locations were drawn from a normal distribution and were either biased to the left or right of the screen center (orange and green probability distributions, P_L_ and P_R_, respectively) or unbiased (center distribution P_C_, in gray). Test circle locations followed an unbiased staircase procedure.

Each trial comprised a series of several ‘prior’ circle stimuli, followed by a single ‘test’ circle stimulus (each discriminated individually, by sequence). The prior stimuli in a given trial were drawn from a specific distribution that was biased (either leftward or rightward) or unbiased. The ‘test’ stimulus was unbiased. This novel paradigm allowed us to examine the rapid effects of short-term biases, in a controlled and interleaved manner. Figure 1 shows the event sequence of a single trial. A fixation cross appeared for 500 ms at the beginning of each trial. This marked the center of the screen, and was presented at the beginning of each trial in order to reduce possible carry-over effects from the previous trial. The fixation cross was not presented again during the rest of the trial in order not to interfere with the priors’ effect being measured. Each circle (prior/test) was presented for 250 ms, and was preceded by a 250 ms delay (blank screen) such that the next circle appeared 250 ms after the participant’s previous response.

We defined the locations of the stimuli (circle center) in the horizontal plane (x) by its visual angle relative to the screen center (x = 0°), such that positive and negative values represent locations positioned to the right and to the left of the screen center, respectively. Locations on the vertical plane were fixed (screen centered). Prior circles locations were drawn randomly from a normal distribution, and were either (on average) biased to the left (mean ± SD: − 3.82 ± 3.82°) or to the right (mean ± SD: 3.82 ± 3.82°) of the screen center, or unbiased (mean ± SD: 0 ± 3.82°). The prior “type” was defined by its bias: ‘left’, ‘right’ and ‘center’, respectively. With this definition, biased priors could still lie to the contralateral side, though with lower probability (~ 16%). Prior stimulus locations were discriminated with high accuracy: 88.3 ± 5.6% correct (mean ± SD; averaged across participants and Conditions #1B, #2 and #3, described below)—i.e., they were relatively easy to discriminate, apart from when they (infrequently) lay close to x = 0°.

Test circle locations were balanced to the right/left (the location sign was randomly selected on each trial), with magnitude (absolute distance from 0°) set using a staircase procedure^31^. This began at a location magnitude of |x|= 6.36° and followed a “one-up, two-down” staircase rule. Namely, location magnitude |x| increased (i.e., became easier) after a single incorrect response, and decreased (i.e., became harder) after two successive correct responses. This staircase rule converges to 70.7% correct performance^32^. Staircase step size followed logarithmic increments, such that location magnitude |x| was multiplied by 0.6 to increase task difficulty and divided by 0.6 to decrease task difficulty. Location magnitude was limited to a maximum of |x|= 25.46°, but this was never reached in practice. Separate staircase procedures were run for each prior type, each comprising 100 trials that were randomly interleaved. Thus, testing all three prior types (right, left and center) comprised 300 trials, and took ~ 30 min, and testing only right/left priors comprised 200 trials and took ~ 20 min. A break was automatically given after every 100 trials, and the experiment was resumed by the participant, when ready.

### Experimental conditions

Six experimental conditions were tested. In four of the six conditions (conditions #1–4), the same stimulus feature (location) was discriminated for both prior and test circles. Hence, the prior decisions were considered relevant to the subsequent test circle perceptual decision. By contrast, in the other two conditions (conditions #5–6), a different feature (color) was discriminated for the prior circles, while location was still discriminated for the test circles. Therefore, the prior decisions were considered irrelevant to the subsequent test circle’s perceptual decision. The results of each such condition were analyzed separately. These six conditions are described below in detail (and summarized in Table 1).

**Table 1:** Experimental conditions.

#	Condition name	Goal	Prior stimulus and task features			
			Biased feature	Discriminated feature (relevant to test)	Circle color	Response keys
1	Baseline (A and B)	Test serial dependence of location discrimination	Location	Location (✔)	White	Left/right
2	Color invariant	Test serial dependence when prior and test stimuli differ in an undiscriminated physical attribute (color)	Location	Location (✔)	Green/purple	Left/right
3	Response invariant	Test serial dependence when discriminations for prior and test stimuli are reported using different responses	Location	Location (✔)	Green/purple	Up/down
4	Response inhibition	Test whether response reporting is necessary for serial dependence to occur	Location	Location (✔)	White	None
5	Alternative feature	Test serial dependence on prior actions that report irrelevant choices	Color	Color (✘)	Green/purple	Left/right
6	Undiscriminated	Test serial dependence on low-level sensory information	Location	Color (✘)	Green/purple	Left/right

✔Indicates that the priors task was relevant to the test task (both were location discrimination).

✘Indicates that the priors task (color discrimination) was irrelevant to the test task (location discrimination).

### Condition #1: “baseline”

In the baseline condition, the prior and test circle stimuli were indistinguishable (white circles). Participants were asked to perform the same task regarding all circles—to discriminate the location of each circle. We tested two variants for this condition: in *Baseline A,* a fixed number of priors (*Q* = 5) preceded the test circle (the parameter *Q* reflects the number of priors on a given trial, and *N* the number of trials, which is also the number of test stimuli). In *Baseline B,* the number of prior circles in each trial was random (*Q* was selected, per trial, according to a discretized Gaussian probability distribution with mean ± SD = 5 ± 2, limited within the range 1–9).We tested all three prior types (right/center/left) in *Baseline A*, but only the two biased prior types (right/left) for the rest of the experimental conditions (*Baseline B* and conditions #2–6), with 100 trials per prior type (i.e., *N* = 300 for Condition #1 *Baseline A* and *N* = 200 for the rest of the conditions). *Baseline A* and *B* were performed by two groups of 20 and 19 participants, respectively. In each variant of this condition, five participants were excluded from the analysis due to poor goodness-of-fit (see *Statistical analysis* section for details regarding the exclusion criterion). The group that took part in *Baseline B* also performed Condition #4 (*Response Inhibition*) during the same experimental session, as a separate block, with block order counterbalanced between participants.

### Condition #2: “color invariant”

In this condition, the same task (location discrimination) was tested for both prior and test stimuli, but the prior circles differed in color. By “color invariant” we mean that color does not help to perform the task (i.e., although color does change, good performance is invariant to this change). The rationale for this condition was to test whether changing a task-irrelevant stimulus feature would affect serial dependence. Also, since color was used in the following conditions to indicate a different task, this condition was required in order to rule out any differences due to color per se. Participants were instructed to discriminate the location of circles, irrespective of their color. This condition was essentially identical to *Baseline B*, except that prior circles were colored (either green or purple) while test circles remained white. All stimuli (priors and test) had equal brightness. The color was randomly chosen for each prior stimulus (50% green and 50% purple), such that the color parameter was unbiased across trials. Twenty-seven participants performed this condition; three participants were excluded from the analysis due to poor goodness-of-fit.

### Condition #3: “response invariant”

In this condition, the same task (location discrimination) was tested for both prior and test circles, but the choices for the prior and test circles were reported using different sets of keys. Like in Condition #2 (*Color Invariant*) prior circles were colored (50% green and 50% purple) and the test circles were white. Participants were instructed to report right and left choices using the ‘up’ and ‘down’ arrow keys (counterbalanced) for colored circles, but using the ‘right’ and ‘left’ arrow keys for white circles. Participants used their second and fourth fingers for the left and right arrow keys and their middle finger for the up and down arrow keys. This condition allowed us to test whether serial dependence ensues even when the actions for reporting choices are dissociated, but the high-level relevance of prior choices is maintained. Twenty-six participants performed this condition; four were excluded from the analysis due to poor goodness-of-fit. Twenty-five of the participants also performed Condition #5 (*Alternative Feature*) in a separate session that took place on a different day (with task order counterbalanced between participants), and 15 agreed to return also for a third session to perform Condition #6 (*Undiscriminated*).

### Condition #4: “response inhibition”

Here, prior and test stimuli were the same (white circles). However, participants were instructed to respond only to the last (i.e. the test) circle in each trial. The number of prior circles followed a random distribution (range 1–9, like *Baseline B*, and all the other conditions except for *Baseline A*). Therefore, the last circle could only be identified in retrospect (when it was not followed by another circle). Hence, the participants needed to discriminate all the circles, but suppress their responses to the prior circles, and respond only to the test circles. This condition allowed us to probe whether choices with unexecuted actions would still elicit serial dependence. This condition was performed by the same participants from Condition #1 (*Baseline B*) in a counterbalanced order. Two participants were excluded from the analysis of this condition due to poor goodness-of-fit.

### Condition #5: “alternative feature”

The aim of this condition was to test whether irrelevant choices (i.e., relating to prior stimulus features other than location) with biased motor responses would elicit serial dependence. Here, participants were instructed to report the color of the circle when it was green or purple (prior circles), but to discriminate location for the white (test) circles. Success in the color task was confirmed by a high percentage of correct responses (mean ± SD = 96.21 ± 2.46%). The prior circle colors in a given trial were biased, with an 84% probability for one color (16% for the other). Trials with prior circles biased green or purple were randomly interleaved in a block. The 84/16 percentage bias was chosen to match the ratio of right/left biased prior locations from the other conditions. Each color was coupled to one of the response keys (right/left arrow), counterbalanced across participants. Participants were instructed to also report the location of the white circle using the same right/left arrow keys. The locations of the prior circles were unbiased and followed the ‘center’ prior type distribution. Thus, the responses to prior stimuli were biased by color choices—irrelevant to the subsequent location discrimination for the test circle. This procedure therefore biased participants’ choices and motor actions in a manner which was irrelevant to the subsequent location discrimination. This condition was performed by 25 participants who also performed Condition #3 (*Response Invariant*) in a different session (counterbalanced). Three participants were excluded due to poor goodness-of-fit and another two because of poor performance on the priors’ task (both below 50% correct, substantially lower than the rest of the participants, who all had above 89% correct).

### Condition #6: “undiscriminated”

The aim of this condition was to test whether biased locations would still elicit serial dependence when not explicitly discriminated. This was done by instructing the participants to report the color for the colored (prior) circles, and location for the white (test) circles. Therefore, by the term “undiscriminated” we mean that location (the feature of interest for the test circles) was not discriminated for the prior circles. The colors of the prior circles were unbiased (50% green and 50% purple). However, their locations were biased (with the same distributions as the other biased conditions). By decoupling the discriminated from the biased stimulus features (color and location, respectively) we were able to probe whether undiscriminated, low-level sensory location information alone biases subsequent location decisions, or whether making an intentional choice regarding the relevant feature is needed for serial dependence to occur. This condition included 38 participants, which consisted of two groups: (i) 15 of the participants who also performed Condition #3 (*Response Invariant*) and Condition #5 (*Alternative Feature*), and (ii) 23 naïve participants, to replicate the results of the former group. Results from the two groups did not differ (in terms of Eq. 2 below; *t*(30) = 1.32, *p* = 0.197, Cohen’s *d* = 0.475), and were therefore merged. Six participants were removed from this condition due to poor goodness-of-fit.

### Data and statistical analysis

Data analysis was performed with custom software using Matlab version R2014a (MathWorks) and the psignifit toolbox for Matlab, version 4^33^. Plots were prepared in Matlab and Figures collated in Corel Draw (version 17). Psychometric plots were defined by the proportion of rightward choices as a function of circle location and calculated by fitting the data with a cumulative Gaussian distribution function. Separate psychometric functions were constructed for each prior type (center, left and right). The goodness-of-fit of the psychometric curves was evaluated using the Likelihood‐ratio based *pseudo‐R‐squared*^34,35^, calculated by the proportional reduction in the deviance of the fitted psychometric model (${D}_{fitted}$) compared to that of the null model (${D}_{null}$):

1 $${{R}^{2}}_{L}=1-\frac{{D}_{fitted}}{{D}_{null}}.$$

Deviance values for the fitted and null model (${D}_{fitted}\mathrm\;{\text{and}}\;{D}_{null},\mathrm{ respectively})$ were calculated using the psignifit toolbox. Only psychometric curves with *R*^2^_*L*_ > 0.5 were used for the final analysis. If any of a participant’s psychometric curves did not reach this level, that participant was removed from further analysis in that condition.

The point of subjective equality (PSE, i.e., the stimulus level with equal probability for making a rightward/leftward choice) was deduced from the mean (*μ*) of the fitted cumulative Gaussian distribution function. The effect of priors on subsequent location discriminations of test circles was assessed by calculating the difference in PSE between left and right prior types, as follows:

2 $$\Delta PSE={PSE}_{left }- {PSE}_{right},$$

where the sign of ΔPSE corresponds to the direction of the serial dependence effect. Positive ΔPSE values indicate an attractive bias (i.e., subsequent perceptual choices are more likely to be the same as prior choices) and negative values indicate a repulsive (or adaptive) bias (i.e., subsequent perceptual choices are more likely to differ from prior choices).

Differences between PSEs of the different prior types (within a condition) was assessed using two-tailed paired *t*-tests, except for *Baseline A,* which included three types of priors (left, center and right) and was therefore assessed using a repeated measures one-way ANOVA with the Bonferroni correction for pairwise comparisons. ΔPSEs were compared between the different experimental conditions using one-way between-subjects ANOVA with pairwise comparisons adjusted with Bonferroni correction. Effect size was estimated by calculating Cohen's *d* and *η*^2^ for *t*-test and ANOVA analyses, respectively.

### Model fits

**Figure 2:**
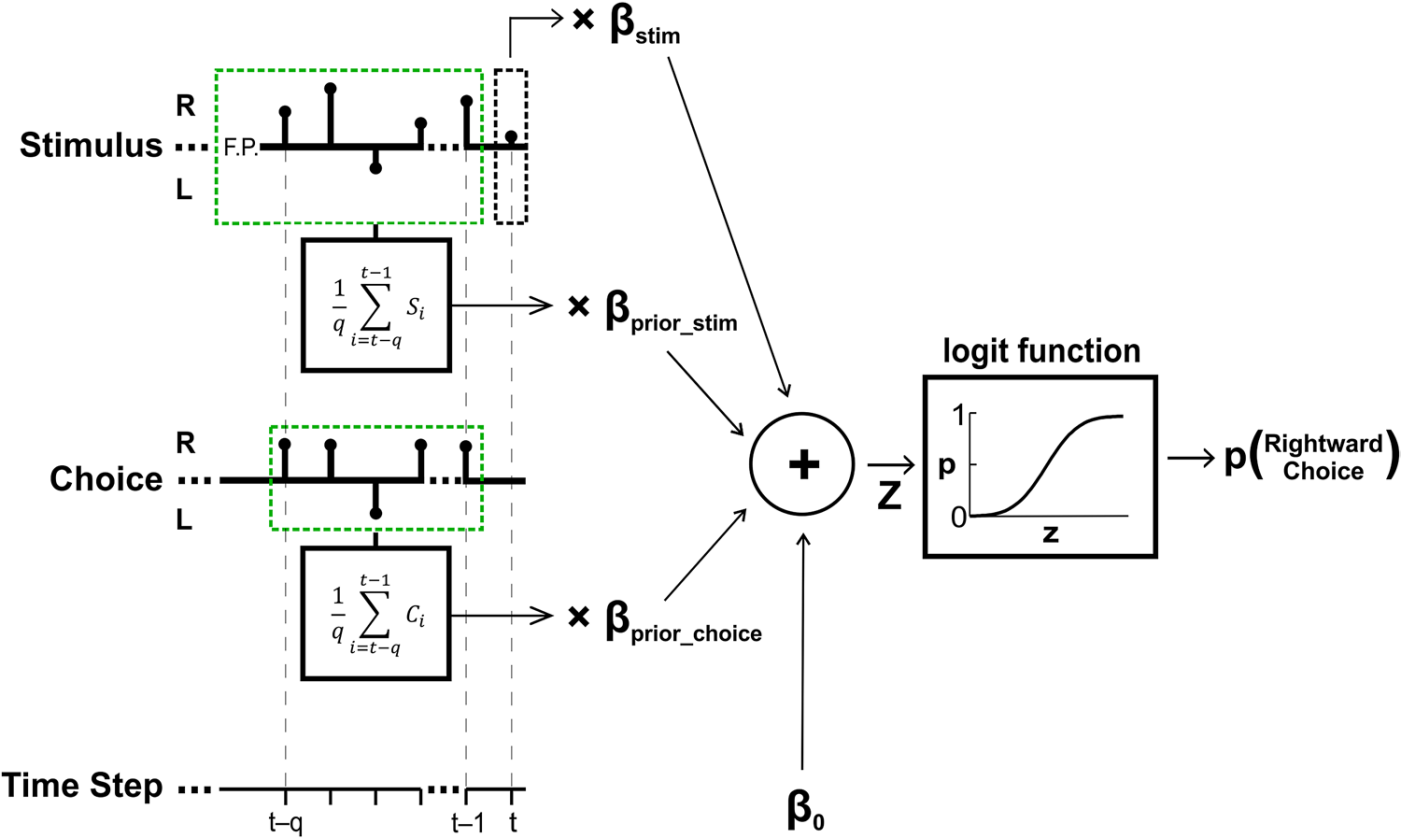
Perceptual decision model schematic. A logistic regression was used to model location discrimination choices based on four predictors (and their respective fitted beta coefficients): (1) current stimulus location (β_stim_), (2) stimulus location history (β_prior_stim_), (3) choice history (β_prior_choice_) and (4) baseline bias (β_0_). Black stems on the stimulus and choice axes mark stimulus (*S*_*i*_) and choice (*C*_*i*_) at time step *i* for an example trial, with test stimulus at *t* (black dashed box), and prior stimuli and choices *q* steps back (for example, priors biased to the right, green dashed boxes). The trial began with a central fixation-point (*F.P.*). The sum of the product of the predictors with their respective coefficients (*z*) is passed through a logistic function to yield the probability of making a rightward choice in response to the test stimulus.

While the ΔPSE (described above) depicts the aggregate behavioral effects of serial dependence, it does not dissociate the effects of prior stimuli from prior choices. Therefore, to further dissect what elements of prior experience influence subsequent choices, we fit the data using a logistic regression model that separated the effects of prior stimuli from prior choices (Fig. 2). We fit the data from five out of the six conditions (it could not be done for Condition #4, *Response Inhibition* because no location discriminations for prior circles were reported).

In this model, each estimated beta coefficient reflects the expected change in the log odds of choosing right for a unit increase in the corresponding predictor variable (e.g., prior stimuli and prior choices) while holding the other predictor variables constant at a certain value. Accordingly, the beta coefficients estimate the specific contributions of the corresponding parameters to the choice, such that the sign and magnitude respectively reflect the direction and size of the effect.

The logistic regression model calculated the probability of making a rightward choice for the current circle stimulus, as follows:

3 $${p}_{t}=\frac{1}{1+{e}^{-{z}_{t}}},$$

where ${z}_{t}$ is a linear combination of different predictors that might affect the current perceptual decision (the specific ${z}_{t}$ used for each model is described below). Choices were binary (right/left; coded by ‘1’ and ‘−1’, respectively), whereas circle locations (i.e., stimulus intensities) were graded along a continuum (with negative and positive values for leftward/rightward locations, respectively). To allow better comparison between the logistic regression weights of these two parameters, circle locations were normalized by the root-mean-square (RMS) of the actual location values presented in the experiment (separately for each experimental condition) such that both parameters had RMS = 1.

We tested and compared four competing models (separately for each experimental condition), that were based on the same logistic regression and differed only by their input parameters:

### M_1_: “no-history” model

This model does not include information from previous trials and was used as a baseline for assessing the added value of incorporating prior information in the following models. The linear combination of predictors used for M_1_ was:

4 $${z}_{t}={\beta }_{0}+{\beta }_{stim}{S}_{t},$$

where ${\beta }_{0}$ represents the observer’s individual baseline bias towards one of the two options (left or right), *S*_*t*_ is the location of the current stimulus and ${\beta }_{stim}$ is its fitted weight.

### M_2_: “stimulus-history” model

M_2_ is like M_1_, but with the addition of prior stimulus information as a predictor:

5 $${z}_{t}={\beta }_{0}+{\beta }_{stim}{S}_{t}+{\beta }_{prior\_stim}\frac{1}{q}{\sum }_{i=t-q}^{t-1}{S}_{i},$$

where $\frac{1}{q}{\sum }_{i=t-q}^{t-1}{S}_{i}$ is the averaged location of the *q* prior stimuli and ${\beta }_{prior\_stim}$ is the weight of this parameter. Setting *q* = 1 (‘1-Back’ analysis) enabled us to investigate the effects of just the previous stimulus and setting *q* = *Q* (‘*Q*-Back’ analysis; where *Q* reflects the number of priors presented on a given trial) enabled us to investigate the aggregate effect of the prior stimuli. In this latter case, our aim was not to quantify the individual effects of each prior stimulus (as is sometimes done, at the cost of additional parameters), but rather to quantify their average effect. This takes advantage of our paradigm design, with consecutively biased priors (interleaved on a trial-by-trial basis), without the need to add more parameters.

### M_3_: “choice-history” model

M_3_ is like M_1_, but with the addition of prior choice (not prior stimulus) information as a predictor:

6 $${z}_{t}={\beta }_{0}+{\beta }_{stim}\mathrm{S}+{\beta }_{prior\_choice}\frac{1}{q}{\sum }_{i=t-q}^{t-1}{C}_{i},$$

where $\frac{1}{q}{\sum }_{i=t-q}^{t-1}{C}_{i}$ is the rightward choice ratio for *q* priors and ${\beta }_{prior\_choice}$ is the weight of this parameter. As before, *q* = 1 was used to investigate the effects of just the previous choice and *q* = *Q* was used to assess the aggregate effect of the prior choices.

### M_4_: “stimulus and choice history” model

Finally, in order to estimate the specific effects of prior stimuli and prior choices on subsequent perceptual decisions, M_4_ included both prior stimuli and prior choices (in addition to the baseline parameters of M_1_):

7 $${z}_{t}={\beta }_{0}+{\beta }_{stim}{S}_{t}+{\beta }_{prior\_stim}\frac{1}{q}{\sum }_{i=t-q}^{t-1}{S}_{i}+{\beta }_{prior\_choice}\frac{1}{q}{\sum }_{i=t-q}^{t-1}{C}_{i}.$$

Here too, *q* = 1 was used to investigate the effects of just the previous prior and *q* = *Q* was used to assess the aggregate effect of the priors.

### Model comparison

We used the Bayesian Information Criterion (BIC) to compare the models. The BIC for a given model (*M*_*i*_) is defined by:

8 $${BIC}_{{M}_{i }}= -2log\left({L}_{i}\right)+ {k}_{i}log\left(N\right),$$

where *N* is the number of observations, in this case, the number of trials (or ‘test’ circles) within an experimental condition. The number of free parameters of *M*_*i*_ is represented by *K*_*i*_, and *L*_*i*_ is its maximum-likelihood. A lower BIC value represents a more likely model^36,37^. BIC values were calculated, per participant, for each of the models. To compare two given models, we calculated the difference between their BIC values (ΔBIC). ΔBIC magnitudes between 2 and 6, between 6 and 10 and > 10 are considered positive, strong and very strong evidence (respectively) for the model with the lower BIC value^37–39^.

## Results

The results from the six conditions of our paradigm (summarized in Table 1), provide a broad view of serial dependence, and specifically implicate task relevance as a key feature for the emergence of serial dependence.

### Location discriminations are biased towards recent choice history

In the standard form of the paradigm (*Baseline*, Condition #1) participants were required to discriminate whether the stimulus (a solid white circle) lay to the right or to the left of the screen center (2AFC). Stimulus locations were biased to the right or to the left for the prior discriminations, followed by unbiased (i.e., balanced) test stimuli. Responses to these test stimuli were sorted by prior bias, analyzed and fit with psychometric curves.

**Figure 3:**
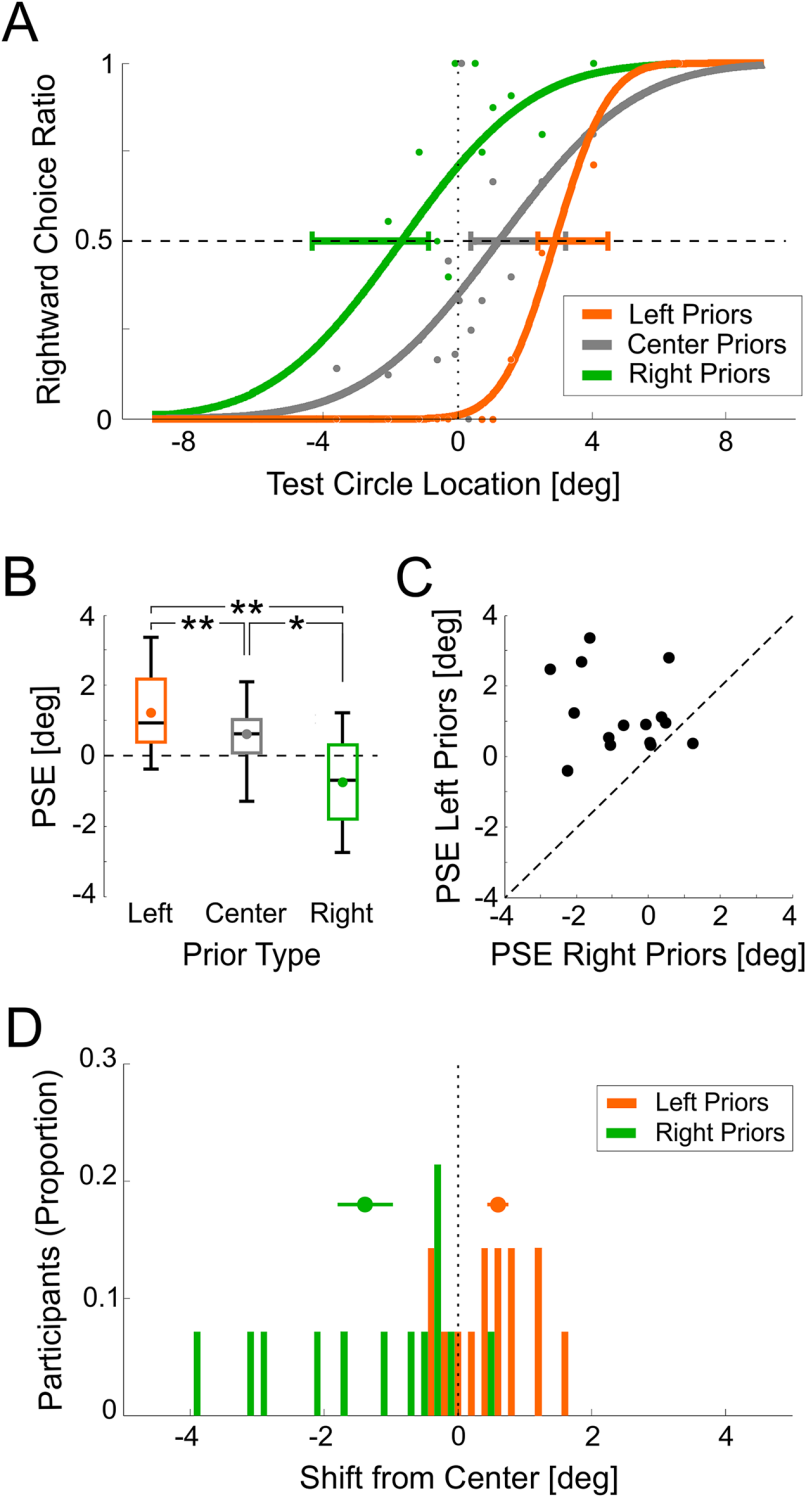
Baseline (Condition #1A) results. (**A**) Psychometric functions are presented for an example participant, sorted by prior type: left, center and right (orange, gray and green, respectively). The data points represent the proportion of rightward choices (for the test circles only) fitted (per prior type) with a cumulative Gaussian distribution function (solid lines). The horizontal dashed line marks the point of subjective equality (PSE; y = 0.5), and the vertical dotted line marks the screen center (x = 0). Horizontal error bars mark 95% confidence intervals of the PSE. (**B**) Summarized PSEs of the group data for the three prior types: left, center and right (orange, gray and green, respectively). The median and range of the data are represented by the black lines and whiskers, respectively. The colored dots represent the respective means. (**C**) PSEs for left vs. right prior types. Each data point represents one participant. The diagonal line marks y = x. (**D**) Distribution of PSE shifts across participants, calculated by subtracting the unbiased (center) PSE from the left and right prior type PSEs (orange and green, respectively). The objective screen center (x = 0) is represented by the vertical black dotted line. Above the histograms, mean ± SEM are presented by the circle markers and horizontal lines, respectively. *p < 0.05, **p ≤ 0.01.

Results from an example participant (Fig. 3A) expose substantial serial dependence. Following rightward biased priors, test stimuli were more likely to be discriminated rightward (green curve). This is best discerned by the value of the psychometric function (> 0.5, i.e., greater likelihood for rightward choices) at the ambiguous stimulus x = 0° (where it crosses the vertical dotted line). This rightward choice bias is also depicted by a negative PSE—the green curve crosses the horizontal dashed line (y = 0.5) at a negative x-value. Similarly, leftward biased priors (orange curve) led to a leftward bias of subsequent test discriminations (and a positive PSE). Responses following unbiased priors (gray curve) lay in between the other two.

Robust serial dependence was also seen across the group (Fig. 3B–D). PSEs differed significantly by prior type (within-subjects one-way ANOVA, *F*(2,28) = 15.03, *p* = 0.000037, *η*^2^ = 0.518). Post-hoc Bonferroni pairwise comparisons revealed significant differences between all three prior types (*p* = 0.01 for the left vs. center, *p* = 0.038, for the right vs. center, and *p* = 0.003 for the right vs. left). A subject-by-subject comparison of PSEs demonstrates high consistency and robustness across all participants but one (14 out of 15 data points lie above the diagonal dashed line; Fig. 3C). Figure 3D presents the distribution of PSEs for leftward and rightward biased priors in relation to each individual’s unbiased/center PSE.

**Figure 4:**
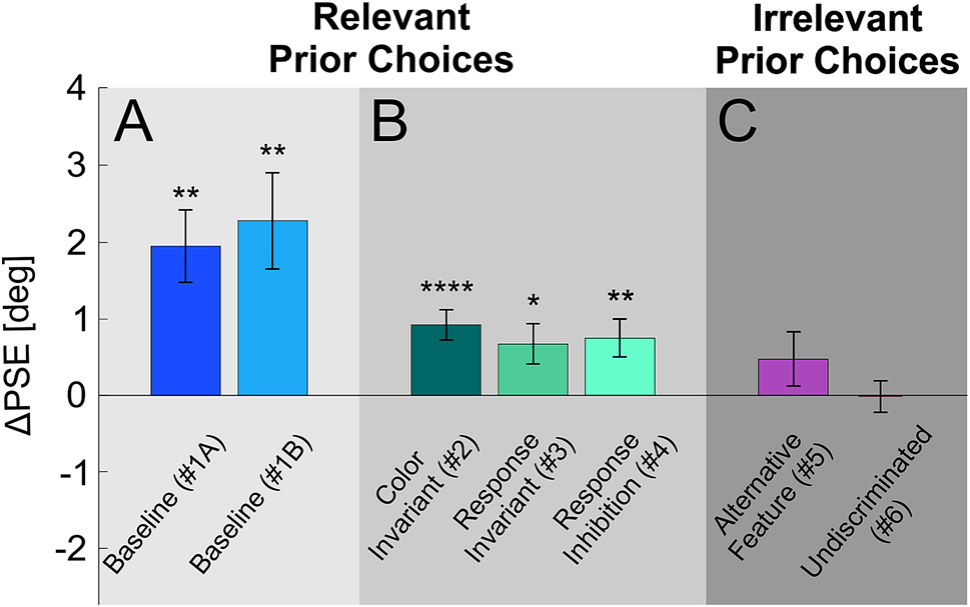
Prior effects across experimental conditions. The PSE difference between left and right prior types (ΔPSE = PSE_Left_priors_ − PSE_Right_priors_) is presented per condition (mean ± SEM). Condition names are presented below the bars (Condition number in parenthesis). Significant shifts are seen for: (**A**) the *Baseline* conditions (blue shades), and (**B**) control conditions in which the task for the prior stimuli was relevant to the test task (green shades). But, not for (**C**) conditions in which the task for the prior stimuli was irrelevant to the test task (pink shades). *p < 0.05, **p < 0.01, ****p < 0.0001.

Next, a second version of the *Baseline* (Condition #1, variant “B”) was run. Variant B differed from variant A in the number of priors (random, between 1 and 9 vs. constant, 5 in variant A), and in testing only the two biased prior types (right and left, without the center/unbiased prior type, which was also tested in variant A). PSE shifts following biased priors were quantified by calculating the difference in PSE for leftward vs. rightward biased priors. Positive ΔPSE values indicate an “attractive” effect. *Baseline* B variant, like *Baseline* A (see above), had significantly positive ΔPSEs (Fig. 4A; *t*(13) = 3.61, *p* = 0.003, Cohen’s *d* = 0.966). Since variant B was better matched methodologically to the control conditions (#2–6), it was used for cross-condition comparisons (below).

### Relevance is key for serial dependence

To test our main hypothesis, we developed five more conditions based on the standard (*Baseline*) condition. These are divided into two main groups: the *relevant* control conditions (Conditions #2–4) and the *irrelevant* control conditions (Conditions #5–6). In the *relevant* control conditions, perceptual discriminations for both the prior and test stimuli were about location. For the *irrelevant* control conditions, prior stimuli discriminated color while test stimuli discriminated location.

In all *relevant* control conditions significant serial dependence was observed (Fig. 4B): Significant PSE shifts were observed in the *Color Invariant* condition (Condition #2), in which prior and test circles differed in terms of color (*t*(23) = 4.7, *p* = 0.000096, Cohen’s *d* = 0.961; Fig. 4B left bar). In the *Response Invariant* condition (Condition #3) discriminations for the prior and test circles were reported using different motor responses (up/down or right/left keys, respectively). Nonetheless, significant PSE shifts were observed (*t*(21) = 2.52, *p* = 0.019, Cohen’s *d* = 0.54; Fig. 4B middle bar). Finally, in the *Response Inhibition* condition (Condition #4) discrimination responses were reported only for the test circles, but were withheld for the prior circles. Here too, significant PSE shifts were still observed (*t*(16) = 3.004, *p* = 0.008, Cohen’s *d* = 0.73; Fig. 4B right bar).

### Irrelevant biases do not lead to substantial serial dependence

In the *irrelevant* conditions, color was discriminated for the prior stimuli, while location was discriminated for the test stimuli. This allowed us to examine the effects of other types of prior biases. Firstly, the effect of motor repetition was tested by generating choice biases for rightward or leftward choices in the priors’ task (Condition #5 *Alternative Feature*). Colors were reported using the same left/right arrow keys as the subsequent location choices for the test stimuli, thereby biasing prior motor responses. Motor biasing led to a small (but not significant) positive serial dependence effect. (*t*(19) = 1.33, *p* = 0.199, Cohen’s *d* = 0.29; Fig. 4C left bar). In the *Undiscriminated* condition (Condition #6), the prior circles’ locations were biased, but participants discriminated color rather than location for the prior circles. In this condition as well, no significant PSE shift was seen (*t*(31) = − 0.1, *p* = 0.92, Cohen’s *d* = − 0.018; Fig. 4C right bar).

### Similarity boosts serial dependence

We further examined whether similarity affects the magnitude of serial dependence. Greater similarity between two events increases our belief that they originate from the same distribution^40^, which might increase serial dependence.

Of the four *relevant* conditions, the *Baseline* condition (Condition #1) had the greatest similarity between prior and test discriminations. All the other *relevant* conditions (#2–4) had specific dissimilarities between prior and test discriminations. Comparing ΔPSEs across these conditions (#1–4; Fig. 4) indeed revealed significant differences (between subjects one-way ANOVA, *F*(3,73) = 4.478, *p* = 0.006, *η*^2^ = 0.155).

First, the contribution of stimulus similarity was assessed by comparing the *Baseline* Condition #1B and *Color Invariant* Condition #2, which differed in color similarity between priors and test stimuli. The *Baseline* Condition #1B had significantly larger ΔPSEs vs. the *Color Invariant* Condition #2 (*p* = 0.03; post-hoc pairwise comparison with Bonferroni adjustment). Therefore, stimulus similarity does boost the effect.

To assess the effects of reporting discriminations using different responses, Condition #3 was compared to the *Color Invariant* Condition #2. No significant difference was seen between the ΔPSEs of these conditions (post-hoc pairwise with Bonferroni *p* > 1). Therefore, serial dependence was still strong even when motor responses were dissociated. Note that in a more nuanced analysis that separates the effects of prior choices from stimuli we do find that using the same motor actions slightly enlarges serial dependence (see section *Choices bias subsequent discriminations* below). Thus, motor similarity seems to magnify, but not to be required for serial dependence.

To assess the effects of withholding responses, *Response Inhibition* Condition #4 was compared to the *Baseline* Condition #1B. These two conditions differed by whether or not responses were reported for the prior stimuli. The *Baseline* Condition #1B had significantly larger ΔPSEs vs. Condition #4 (*p* = 0.02, post-hoc pairwise comparison with Bonferroni adjustment). Although the same participants took part in both conditions, for consistency with the other comparisons in this section a between-subjects comparison was used (a within-subjects comparison provided similar results: *p* = 0.016). Therefore, actively reporting prior discriminations boosts serial dependence.

### Choices bias subsequent discriminations

What aspect of prior discriminations drives serial dependence—prior choices, prior stimuli, or a combination of the two? To answer this question, we fit the data on a trial-by-trial basis with a logistic-regression model that could account separately for the effects of prior choices and prior stimuli (see Fig. 2 and Methods section *Model fits* for details). For comparison, we fit the logistic regression with four different combinations of parameters. All four had two standard parameters (to account for the effects of the current stimulus and a baseline bias), and differed by whether or not they also took into account prior choices and/or prior stimuli, as follows: (M_1_) no history (no prior choice/stimulus information), (M_2_) stimulus history only, (M_3_) choice history only or (M_4_) both stimulus and choice history. Model fits were performed per participant, and condition (except for Condition #4, which was not fit because choices were not reported for prior stimuli).

**Figure 5:**
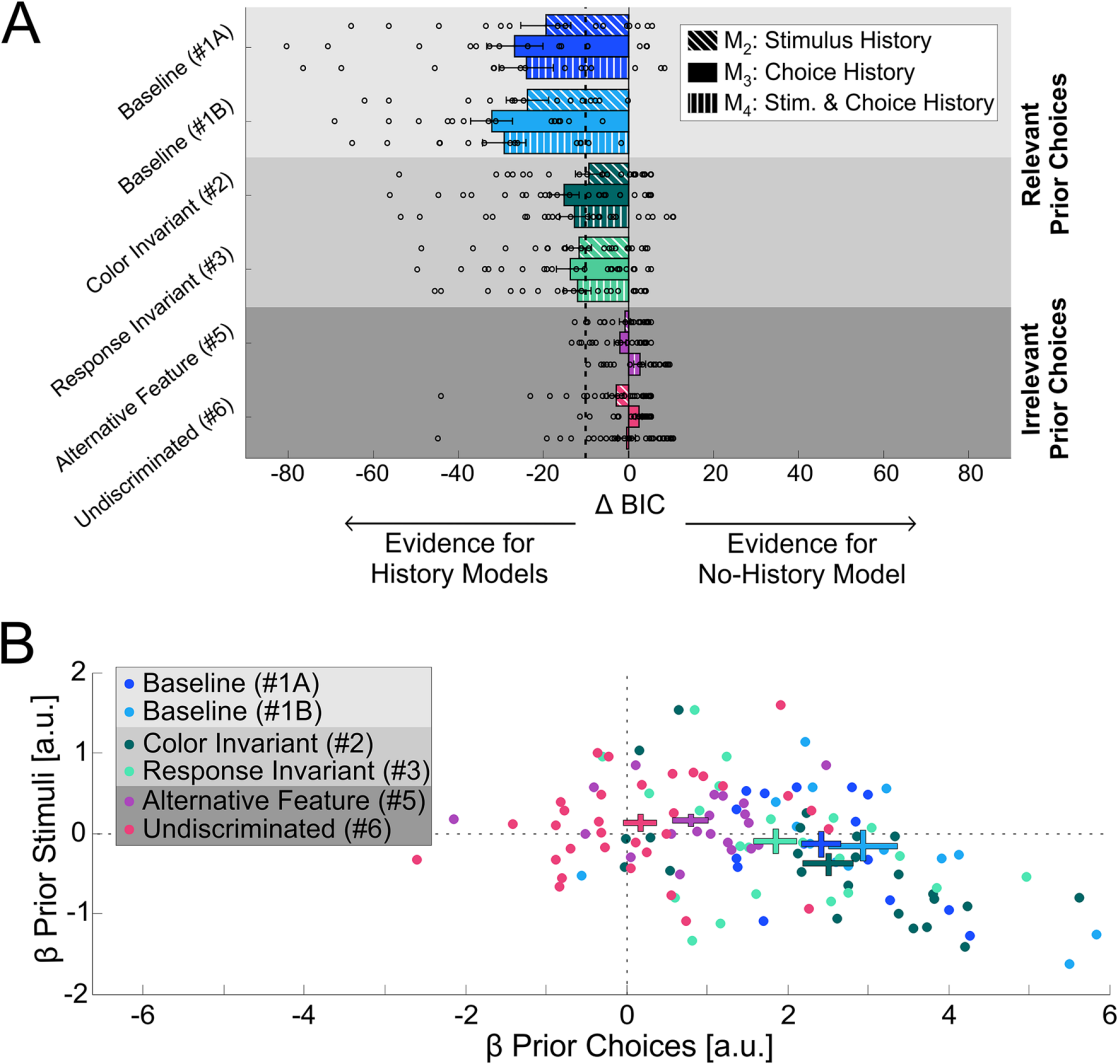
Model Comparisons. (**A**) The Bayesian information criterion (BIC) is presented for the three history models in relation to the ‘no history’ model, across experimental conditions. ‘Stimulus history’, ‘choice history’ and ‘stimulus and choice history’ models are represented by the diagonal-stripe, filled and vertical-stripe textured bars (ordered: top, middle and bottom within each condition), respectively. Negative ΔBIC values (ΔBIC = BIC_M*i*_—BIC_M1_; where M_1_ represents the ‘no-history’ model and M_*i*_ represents one of the ‘history’ models, *i* = 2, 3 or 4) indicate more evidence for the ‘history’ model. ΔBIC < − 10 is considered very strong evidence for the history model (the vertical dotted line marks ΔBIC = − 10). Circles represent ΔBIC values of individual participants, and bars and error bars mark the mean ± SEM. Choices were better explained by models with history for: the baseline conditions (light gray background) and control conditions (medium gray background) in which the task for the prior stimuli was relevant to the test task, but not for conditions in which the task for the prior stimuli was irrelevant to the test task (dark gray background). (**B**) Beta coefficients for the prior stimulus vs. prior choice (from the ‘stimulus and choice’ history model M_4_) are plotted for each participant (data points), per condition (color coded). Crosses represent the mean ± SEM per condition.

To quantify the value of including prior information, we calculated the difference in Bayesian Information Criterion (ΔBIC) between each of the history models (M_2–4_) vs. M_1_ the no-history model (Fig. 5A; diagonal-stripe, filled and vertical-stripe bars, for M_2_, M_3_ and M_4_, respectively). For the *relevant* conditions (Fig. 5A, light and medium gray backgrounds) ΔBICs are large and negative, indicating better model fits with history (the vertical black dashed line at ΔBIC = − 10 marks very strong evidence for the models with history^36–38^).

By contrast, for the *irrelevant* conditions (Fig. 5A, dark gray background), ΔBIC values are small, indicating no clear preference for the models with history. The results presented here used ‘*Q*-back’ model fits (i.e., all the priors in a trial). Using ‘1-Back’ fits (including only the most recent prior) also showed a preference for the history models in the relevant conditions, but with smaller ΔBIC values (ΔBIC < 8 compared to the large ΔBIC values seen in Fig. 5A).

Comparing between the history models in the *relevant* conditions (Fig. 5A, light and medium gray backgrounds) shows better performance for M_3_ (choice history only) vs. M_2_ (stimulus history only) or even M_4_ (both stimulus and choice history). We further compared the beta coefficients of prior stimulus vs. prior choice from M_4_, and found that prior choices had a larger influence vs. prior stimuli (Fig. 5B). The data lie off to the right of the y-axis (indicating large prior choice effects), but not far from the x-axis (indicating minor effects of prior stimuli). This is especially evident in the *relevant* conditions for which the prior choice beta coefficients were significantly positive (one sample *t*-test, *t*(14) = 9.75, *t*(13) = 6.91, *t*(23) = 7.93 and *t*(21) = 6.88, for Conditions #1A, 1B, 2 and 3, respectively, *p* < 0.00003 for all, after Bonferroni correction for multiple comparisons). By contrast, their prior stimulus beta coefficients were small and statistically insignificant (*t*(14) = − 0.8, *t*(13) = − 0.8, *t*(23) = − 2.73 and *t*(21) = − 0.66, for Conditions #1A, 1B, 2 and 3, respectively, *p* > 0.28 for all, after Bonferroni correction for multiple comparisons).

Comparing prior choice coefficients across all conditions revealed that they were significantly smaller in the *irrelevant* vs. the *relevant* conditions (between subjects one-way ANOVA, *F*(5,121) = 16.595, *p* = 0.0000001, *η*^2^ = 0.407). Post-hoc pairwise comparisons between each of the *irrelevant* conditions vs. each of the *relevant* conditions all revealed significant differences (Fig. 5B; *p* < 0.004 for all, with Bonferroni adjustment). Prior choice coefficients were small but nonetheless significantly positive for Condition #5 (purple cross; *t*(19) = 3.54, *p* = 0.048 after Bonferroni correction for multiple comparisons), and not statistically significant for Condition #6 (pink cross; one sample *t*-test, *t*(31) = 0.84, *p* = 0.4, not Bonferroni corrected). Prior stimulus coefficients were small and not significant for both the *irrelevant* conditions (one sample *t*-test; *t*(19) = 1.95, *p* = 0.065 and *t*(31) = 1.23, *p* = 0.23 without Bonferroni correction, for Conditions #5 and #6, respectively).

**Figure 6:**
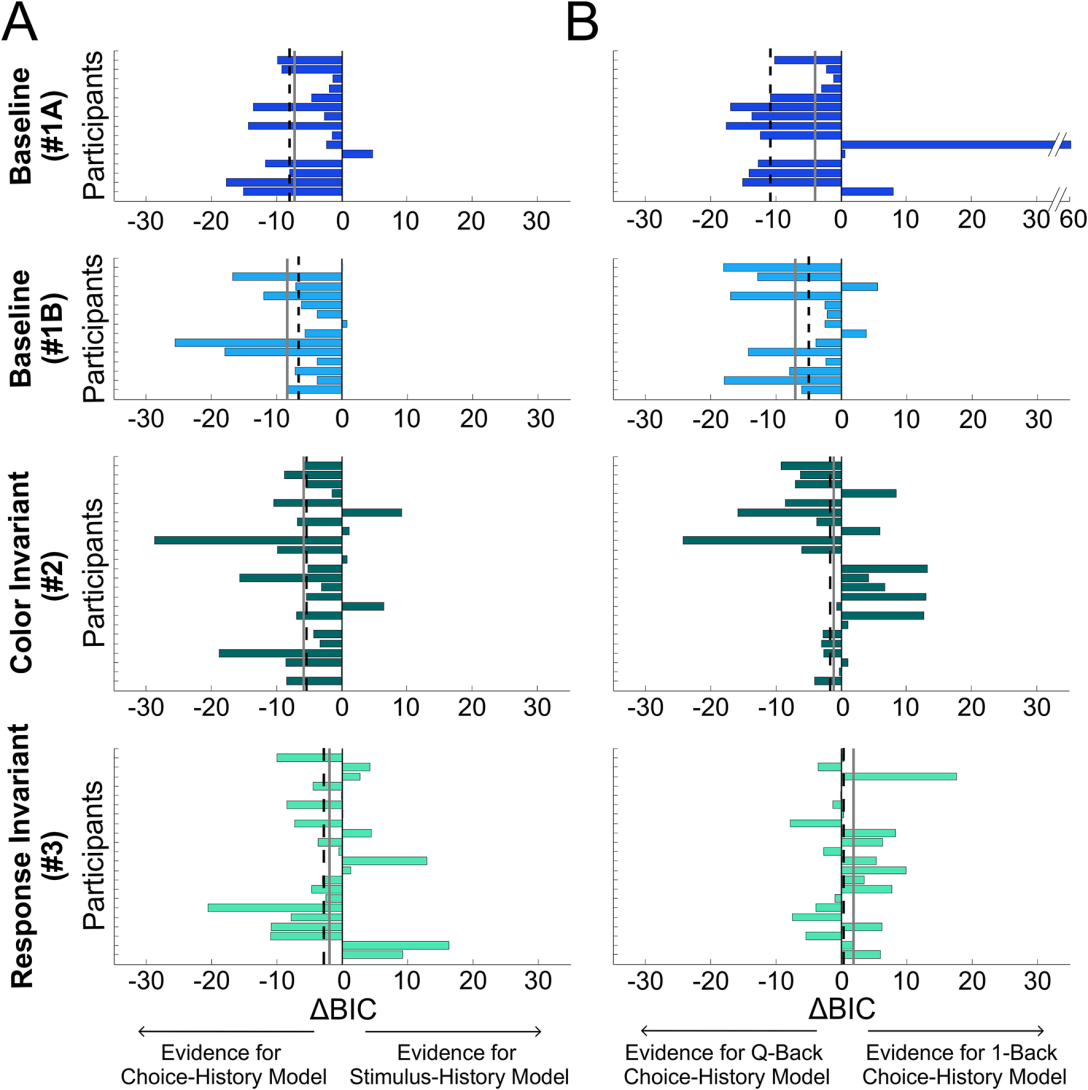
Choice vs. stimulus history comparison. (**A**) Comparison between ‘choice-history’ (M_3_) and ‘stimulus-history’ (M_2_) models for each participant in the four ‘relevant’ conditions. Negative ΔBIC values mean more evidence for the ‘choice-history’ (vs. ‘stimulus-history’) model. Each bar represents the ΔBIC value for a single participant. The gray solid and black dashed lines represent the mean and median, respectively. (**B**) Comparison between ‘Q-Back’ and ‘1-Back’ choice-history models for the four ‘relevant’ conditions. Negative ΔBIC values mean more evidence for the ‘Q-Back’ (vs. ‘1-Back’) model.

Calculating ΔBIC between the choice history model (M_3_) and the stimulus history model (M_2_), on a subject-by-subject basis, replicated the observation that prior choices affect subsequent decisions more than prior stimuli. *Baseline* Conditions #1 A and B had large, negative ΔBIC values on average (negative for 90% of participants; Fig. 6A, top two rows). In Condition #2 ΔBIC values were still negative on average but slightly less so (negative for 75% of participants; Fig. 6A, third row). And, in Condition #3 ΔBIC values were less negative (negative for 54.5% of participants; Fig. 6A, bottom row).

### Accumulation effect of similar discriminations

We next asked whether serial dependence is enhanced by a series of prior events or whether it is dominated by the most recent one. Therefore, we examined whether the observers’ responses to test stimuli are better predicted by the aggregate effect of the *Q* prior choices in the current trial (‘*Q*-Back’) or by just the most recent one (‘1-Back’). This was quantified by calculating the ΔBIC between *Q*-Back and 1-Back model fits. The large negative ΔBIC values for the *Baseline* Condition #1 (Fig. 6B; top two rows) show a *Q*-Back preference, reflecting an accumulation effect of prior choices (negative ΔBIC values on average, and for 80% of participants). Here too, this effect decreased as similarity of the priors decreased. ΔBIC values were on average closer to zero for Conditions #2 and #3 (Fig. 6B, third and fourth row, respectively) no longer showing a preference for *Q*-Back.

An accumulation effect of priors might suggest that a larger number of priors should lead to an increased serial dependence effect. To test this hypothesis, we divided the trials into two groups—those with *less* (*Q* < 5, i.e.: 1–4) or *more* (*Q* ≥ 5; i.e.: 5–9) priors, and compared their ΔPSEs, for each of the *relevant* conditions (#1–4). Indeed, we found that the difference between *less* or *more* priors was dependent on condition (*F*(3,73) = 2.863, *p* = 0.043, *η*^2^ = 0.102, two-way ANOVA interaction). Specifically, only for Condition #1, trials with *more* priors elicited stronger serial dependence (ΔPSE mean ± SD = 2.12 ± 0.33°) compared to trials with *less* priors (1.19 ± 0.32°; *p* = 0.005; post-hoc pairwise comparison with Bonferroni adjustment). No significant differences were found in the rest of the conditions (*p* > 0.28 for all; post-hoc pairwise comparisons with Bonferroni adjustment). These results as well indicate that greater similarity of prior events leads to greater accumulation effects.

### Reduced carry-over to the next trial

The test stimulus (last stimulus presented in a trial) was unbiased on average and converged on locations close to zero (due to the staircase). In itself, this should reduce carry-over effects from the preceding trial’s priors to the next trial (but might not eliminate them, due to an accumulation effect). Thus, to further reduce any carry-over effects, each trial began with a fixation point. This also slightly increased the time between the last (test) stimulus of the previous trial and the first prior of the next trial. To validate that carry-over effects from the preceding trial’s priors to the next trial were small, we tested whether discriminations of the first prior of a trial (after the fixation point) were biased by perceptual history from the preceding trial (before the fixation point). Responses to these ‘first prior’ stimuli were sorted according to the prior bias from the preceding trial, and analyzed and fit with psychometric curves (in the same manner presented above for the test stimuli). This analysis was performed on the data from Condition #1 (*Baseline* variant B) because these data had large serial-dependence effects with accumulation, and in this condition, prior and test circles were indistinguishable to the participants, and the number of priors was random (so the appearance of a fixation-point could not be anticipated).

We found no significant difference between PSEs when separating the data by the prior type (left/right) of the preceding trial (paired *t*-test, *t*(13) = − 1.83, *p* = 0.09, Cohen’s *d* = − 0.49). Assessing the effects of the preceding trial on discriminating the first prior of the next trial using the logistic regression model (M_4_) showed a reduced effect of prior choices compared to that described for the test discrimination (prior choice beta coefficients mean ± SD = 1.31 ± 1.59° vs. 2.94 ± 1.59°; paired *t*-test *t*(13) = − 3.5, *p* = 0.004, Cohen’s *d* = − 0.94). However, this effect of prior choices was nonetheless still statistically significant (one-sample *t*-test, *t*(13) = 3.08, *p* = 0.036, after Bonferroni correction). Hence, the effects of prior choices were reduced (by more than half) but not eliminated. Like above (for the test circles), prior stimulus beta coefficients were not statistically significant (mean ± SD = − 0.21 ± 0.84°, one-sample *t*-test, *t*(13) = − 0.95, *p* = 0.36, without Bonferroni correction). The finding of a significant (albeit small) effect that persists even beyond the presentation of a fixation point suggests that a boundary shift alone does not account for the results.

## Discussion

In this study, we found strong serial dependence between sequential perceptual decisions, only when they were relevant to one another—namely, when the same perceptual feature (location in our experiment) was discriminated for both prior and current stimuli. Even when a feature was biased in the series of prior trials, it did not bias subsequent decisions about that feature when it was not discriminated in the prior trials—attesting to the pivotal role of relevance of prior choices in serial dependence. Our model fits showed that serial dependence was best explained by prior choices rather than prior stimuli, and that this effect is aggregated over several prior trials. Furthermore, while serial dependence required relevance of prior choices, it was modulated by similarity of sensory attributes (color) and action-related attributes (motor response execution).

The observed serial dependence in location discrimination could reflect: (a) a shift in the perceived locations of the stimuli, (b) a shift in the estimated boundary (screen center), (c) a change in stimulus–response mapping, or a combination of these factors. Our finding that the effect of prior choices was still significant (albeit reduced) even after the presentation of a fixation point argues against a pure boundary shift explanation. Also, a series of biased stimuli (e.g. to the right), might be expected to draw the boundary estimate towards the same side (also to the right), which would lead to a choice bias to the opposite side (to the left)—opposite to our results. This suggests that the observed serial dependence does not reflect only a shift in the boundary estimate, and, at least in part, reflects a change in stimulus–response mapping.

Our finding that relevance is key for serial dependence corroborates previous studies. For example, it was recently shown that serial dependence is markedly reduced when attention to the relevant feature is lacking in the preceding stimulus^41^. Our results extend these findings by providing evidence that this occurs in an accumulative manner (not just to a single prior stimulus) and by showing how it is modulated by low-level similarity between prior and test stimuli. Our findings are also in line with another recent study that found no serial dependence when prior discriminations were about the variance of a stimulus value and subsequent discriminations about its mean^26^, considering variance and mean to reflect different features of the stimulus. However, in that study, the different discriminations were reported using different motor actions. Therefore, it is unclear whether the lack of serial dependence resulted from dis-similarity in motor execution or the lack of relevance of prior discriminations. Our results address this by showing that motor similarity in itself does not trigger substantial serial dependence between irrelevant decisions. This is in line with Braun et al.^42^, who also found that choice history bias is not explained by motor responses, and with Cicchini et al.^18^, who demonstrate little contribution of motor responses to serial dependence. Additionally, we found that serial dependence takes place even when priors are not reported at all^43^.

Presumably contrary to our findings, Fornaciai and Park^28,29^ found serial dependence in numerosity perception, even when participants were told to ignore the prior stimuli, which were not relevant to the subsequent task. Their conclusion that task-irrelevant stimuli do elicit serial dependence might seem, at face value, to contradict our results (and those of Suárez-Pinilla et al.^27^). However, closer inspection reveals that the results are actually consistent—the difference lies in the definition of “relevant.” In the numerosity task, the prior stimuli were indeed objectively irrelevant since they contributed no information to solving the subsequent task. However, this is the case for almost all psychophysics tasks in which stimuli are independent over trials. Yet serial dependence is still observed, even though participants are instructed to discriminate just the stimulus presented (with the obvious implication that prior information is not relevant). By contrast, we use the term ‘relevance’ here to reflect an implicit and subjective inference of relevance—i.e., when the same task is performed in series, the observer might behave *as if* the prior stimuli are statistically relevant. Given that consecutive events in the world are generally not independent, this may reflect a valid evolutionary skill.

Furthermore, in the numerosity experiments^28,29^ the prior stimuli lay embedded within a sequence of the same stimuli that were repeatedly discriminated. Thus, it is probable that the prior stimuli were also discriminated to some degree, even if participants were instructed to ignore them. The authors’ subsequent finding that attention to the prior stimuli is necessary to elicit serial dependence supports this view. Accordingly, those experiments may be more similar to our Condition #4 (*Response Inhibition)*. In this condition, location of prior stimuli was not reported, but still had to be discriminated (otherwise the task could not be accomplished). This result is in line with previous experiments that also demonstrate serial dependence in the absence of motor actions^6,43,44^. Notably, the effect for Condition #4 (*Response Inhibition*) was in the same direction (positive/ attraction, as opposed to a negative/ adaptation effect) and of similar magnitude as the other *relevant* conditions. Therefore, we suggest that prior perceptual decisions that are relevant, but not reported can still elicit serial dependence.

Our model analyses showed that perceptual decisions in our study were biased specifically by, and towards, prior choices, and not prior low-level sensory information. This is in line with other recent studies that implicate prior choices as the cause for serial dependence^24,25,42^. This is most clearly demonstrated in the *Undiscriminated* condition, in which serial dependence regarding location did not emerge when stimulus location was biased but not discriminated. As such, serial dependence seems to differ from visual priming, which primarily emanates from the stimulus itself^42^. It should be noted that a correlation between choice and stimulus information is inevitable, which could make it more difficult to discern the separate effects of prior stimuli and choices. Nevertheless, in this task stimuli and choices did differ from one another for two reasons. First, stimuli were graded, whereas choices could only have binary values (± 1). Second, observers make mistakes, especially for stimuli that lie close to the screen center. These differences allowed the models to identify prior choices as the primary factor of serial dependence—supported by better performance of the choice history model (M_3_) vs. the stimulus history model (M_2_) and a comparison of prior choice vs. prior stimulus coefficients from the combined stimulus and choice history model (M_4_).

The fact that choices rather than stimuli triggered serial dependence, may shed light on the role of spatial attention in serial dependence. All the conditions reported here, required participants to allocate some degree of spatial attention, to be able to see the stimuli in the task. Nonetheless, this form of spatial attention in itself did not trigger serial dependence, not even when the location of priors was systematically biased. A distinction should therefore be made between two forms of spatial attention. The first pertains to seeing the stimuli that needs to be discriminated in its location, and is triggered in a bottom-up manner by the stimuli. The second relates to top-down mechanisms of prioritizing location as the specific feature to be discriminated. In the context of the location discrimination task, this high-level type of spatial attention may be the basis for presumed relevance, and was critical for serial dependence to emerge.

In addition to our finding that prior choices (and not low-level sensory information) trigger serial dependence, we also found that the effect of prior choices was modulated both by sensory information of color, as well as by motor responses. Specifically, serial dependence was reduced when the stimuli differed in color and when motor responses were either withheld, or when different actions were used to report choices. How does similarity in sensory and motor processes contribute to serial dependence? With regards to motor responses, the action-related boosting of serial dependence does not seem to result from general processes activated by motor actions, such as enhanced post-perceptual memory^45,46^ or enhanced attention to events that are acted upon, because serial dependence was smaller when different actions were used to report choices. A more probable suggestion is that similarity binds events through categorization, such that similar events are labeled under the same category^40^. Prior events from the same (or close) category would be more likely to share their statistics, making them more useful to predicting upcoming stimuli.

Indeed, a study by Petzold and Haubensak^47^ has shown that when participants were instructed to relate to stimuli with different colors as belonging to different categories, a serial dependence effect emerged only within category. But when participants were explicitly asked to ignore stimulus color and relate to all stimuli as belonging to the same category, the serial dependence effect emerged within all types of stimuli. These findings support the idea that low-level similarities may contribute to higher-order mechanisms such as categorization and grouping of events. Taken together, our results point to an interesting interaction between relevance of prior choices and other aspects of the prior stimuli and actions for serial dependence: that is, choice relevance is key in generating serial dependence, which is then modulated by low-level stimulus similarity and motor execution. This is in line with another recent study that showed stronger serial dependence when contextual features were the same across trials^48^.

Further research is required to understand how neural activity supports serial dependence. One suggestion is that attraction to prior choices might result from residual activity in neuronal populations that encode perceptual choices, and therefore increase the probability for making the same choice again^49^. This theoretical mechanism is in accordance with animal studies that show priors and choice history activity in posterior parietal cortex (PPC)^50,51^. Also the prefrontal cortex (PFC) encodes goals and outcomes of previous trials^52^. Specifically, the ventro-lateral PFC has been shown to encode prior stimuli, choices and outcomes, and stimulation of this brain region biases subsequent behaviors^53^. Furthermore, choices, actions and stimulus signals are mixed in both PPC^54^ and PFC^55^, which makes these brain regions suitable candidates to integrate information across these heterogeneous factors. Thus, it is possible that PPC and PFC offer a high-level substrate to assess and compare prior choice relevance, in light of low-level stimulus and action conjunctions. They may bias subsequent choices accordingly via the basal ganglia^56,57^.

In conclusion, our findings highlight high-level choice relevance as a critical trigger for serial dependence. Using a novel paradigm with unbiased test stimuli we show that serial dependence aggregates over a series of biased priors, but occurs only when the relevant feature was discriminated. We further show that although low-level (sensory and motor) features do not themselves trigger serial dependence, greater low-level similarity boosts the effect. Therefore, we suggest that serial dependence reflects an active mechanism in the brain, in which only relevant prior choices are used to predict and bias ongoing perceptual decisions.
